# Ethics of Short-Term Experiences in Global Health: Engaging Skeptics of Change

**DOI:** 10.5334/aogh.2529

**Published:** 2019-06-17

**Authors:** Michael Rozier

**Affiliations:** 1 Department of Health Management and Policy, Saint Louis University School of Public Health, US

## Many Tools to Fix the Problem

Short-term experiences in global health (STEGHs) have rightly been the subject of increased scrutiny [1], and many scholars have provided critiques and recommendations as to how they might be improved [2,3]. Rowthorn and colleagues present the most robust analysis of where STEGHs move beyond being unethical to also being illegal [4]. Raising awareness of how STEGHs can violate the law is an important approach to correcting unethical behaviors. Another tool, as the authors also suggest, is the soft power of professional norms and global standards, which may involve “naming and shaming” violators or creating accreditation programs to STEGHs. In addition, I would suggest that those who wish to change behavior must directly engage with those who may not entirely agree that these experiences need significant changes.

## Engagement with Skeptics of Change

Despite the fact that many will be horrified by the anecdotes shared by Rowthorn and colleagues, for many, the solution is not immediately obvious. This is because skeptics of change hold competing interests that create, in their minds, genuine dilemmas. Below, I raise common objections to the idea that STEGHs need wholesale change and offer a very brief response to each.

(1) *Some care is better than no care. Although it is not ideal to have people performing outside the scope of their formal training, patients and the community are better off than they otherwise would be*.

This is the most common defense of STEGHs in their current form. And it relies on a narrow utilitarian calculation that negative patient outcomes are outweighed by the positive ones. I have never seen a credible empirical assessment of this claim [5]. And the often-hidden negative outcomes, such as creating a belief among host communities that local providers are not effective or providing short-term solutions to long-term health problems, are difficult to calculate. But even if the net utility was positive, such a position fails to consider other values we hold dear in health care. For example, this approach conditions providers and teaches students that unequal treatment of patients is acceptable and that the poor are fortunate to get whatever they can, even if it violates standards of care. That cannot help but shape the way they see the world.

On the other hand, the above claim is ethically acceptable in times of emergency. Natural or other disasters create situations wherein those without proper training do whatever good they can, even if it causes some harm along the way. Some may claim that the lack of health care in areas visited by STEGHs is equivalent to an emergency situation, thus justifying extraordinary action. However, most STEGHs have long-term, established relationships with local communities. If they had invested in public health infrastructure, health education, and health profession training, the emergency conditions would have long ago been resolved.

(2) *If host communities did not want STEGHs acting as they do, they would enforce different standards. Since host communities accept actions of STEGHs, that should be enough*.

I am sympathetic to this argument because I believe host communities should generally be given the power to deem what is acceptable and what is not. However, this position generally ignores two things. First, as Rowthorn and colleagues observe, many countries have already stated what is acceptable and what is not. Many of them have licensure laws that volunteers ignore. A country’s lack of the capacity to enforce its laws does not mean its will should be dismissed. Second, this claim ignores the power differential that prevails between volunteers and host organizations. Many communities rely on the funds spent by volunteers – on lodging, transportation, food, souvenirs, and more. And many host organization’s employees are dependent upon a steady stream of volunteers for their livelihood. Even more, in many cultures, being good hosts is a central value and so it would be anathema to critique a guest, even when their actions are harmful. Therefore, this argument can only be credible in situations where we are confident that power and cultural norms are not shaping the dynamic between volunteers and host organizations.

(3) *These are important experiences for volunteers and often lead to life-long commitments to service in the global south. If we make it less attractive, we reduce the connections that lead to long-term relationships*.

We *should* provide opportunities that engender solidarity across communities. Yet instead of solidarity, many STEGHs establish a disposition of volunteer as savior or tourist. This is especially true when medical professionals model unethical or illegal behavior for students. We should ensure volunteer experiences are creating the habits that we want replicated over the long-term, which includes following best practices even when they seem inconvenient [6]. Otherwise, we might be encouraging life-long commitments, but they will not be the kind of commitments that help transform the communities in need.

Many of these experiences provide greater benefit to the volunteers than to the communities served. Ignoring that reality is, in part, what makes these experiences so troubling. In my opinion, STEGHs would have far less to prove if they confronted the false narrative that they are primarily altruistic.

## Conclusion

Many people agree with the three statements above and critics of STEGHs dismiss them at the expense of making change more likely. The simple fact that so many organizations and individuals continue to behave in defiance of the law and in violation of ethical norms means that there are countervailing values that must be dislodged. Engaging with these skeptics is an important tool, alongside those suggested by Rowthorn and colleagues, to making STEGHs not only comply with legal standards, but also align with the ethical norms of health care practice.

## References

[1] Bauer I. More harm than good? The questionable ethics of medical volunteering and international student placements. Trop Dis Travel Med Vaccines. 2017; 3: 5 DOI: 10.1186/s40794-017-0048-y 28883975 PMC5531079

[2] Roche SD, Ketheeswaran P and Wirtz VJ. International short-term medical missions: A systematic review of recommended practices. Int J Public Health. 2017; 62(1): 31–42. DOI: 10.1007/s00038-016-0889-6 27592359

[3] Martiniuk AL, Manouchehrian M, Negin JA and Zwi AB. Brain gains: A literature review of medical missions to low- and middle-income countries. BMC Health Serv Res. 2012; 12: 134 DOI: 10.1186/1472-6963-12-134 22643123 PMC3474169

[4] Rowthorn V, Loh L, Evert J, Chung E and Lasker J. Not above the law: A legal and ethical analysis of short-term global health experiences. Annals of Global Health; 2019. 10.5334/aogh.2451 PMC6634331 31225956

[5] Sykes KJ. Short-term medical service trips: A systematic review of the evidence. Am J Public Health. 2014; 104(7): e38–48. DOI: 10.2105/AJPH.2014.301983 PMC4056244 24832401

[6] Catholic Health Association. Guiding principles for conducting international health activities. 2015 https://www.chausa.org/internationaloutreach/guiding-principles. Accessed 5 May 2019.

